# Insights into the role of bioactive plants for lambs infected with *Haemonchus contortus* parasite

**DOI:** 10.3389/fvets.2025.1566720

**Published:** 2025-03-12

**Authors:** Michaela Komáromyová, Daniel Petrič, Klára Demčáková, Matej Leško, Klaudia Čobanová, Michal Babják, Alžbeta Königová, Tetiana Kuzmina, Sylwester Ślusarczyk, Paulina Izabela Fortuna, Anna Łukomska, Pola Sidoruk, Adam Cieslak, Zora Váradyová, Marián Várady

**Affiliations:** ^1^Institute of Parasitology of Slovak Academy of Sciences, Košice, Slovakia; ^2^Institute of Animal Physiology of Centre of Biosciences of Slovak Academy of Sciences, Košice, Slovakia; ^3^University of Veterinary Medicine and Pharmacy in Košice, Košice, Slovakia; ^4^I. I. Schmalhausen Institute of Zoology NAS of Ukraine, Kyiv, Ukraine; ^5^Department of Pharmaceutical Biology and Biotechnology, Wroclaw Medical University, Wrocław, Poland; ^6^Omics Research Center, Wroclaw Medical University, Wrocław, Poland; ^7^Department of Preclinical Sciences and Infectious Diseases, Poznan University of Life Sciences, Poznań, Poland; ^8^Department of Animal Nutrition, Poznan University of Life Sciences, Poznań, Poland

**Keywords:** antioxidant status, chicory, histology, lambs, parasitological status, phytochemicals

## Abstract

Bioactive plants provide therapeutic and prophylactic effects to ruminants. We determined the effect of grazing on natural meadow grassland enriched with experimentally sown chicory (*Cichorium intybus*) on parasitological status, pasture larval infectivity, antioxidant parameters, and the histology of abomasal tissue in lambs experimentally infected with the parasitic gastrointestinal nematode (GIN) *Haemonchus contortus.* We also qualitatively identified the main polyphenols in the meadow grassland and phenolic metabolites in the feces of the lambs. Sixteen lambs were orally infected with approximately 5,000 infective larvae (L3) of *H. contortus*. The lambs were divided into two groups: lambs grazing on a plot consisting exclusively of meadow pasture which serves as control group and lambs grazing on a plot where approximately 25% of a meadow grassland was reclaimed with chicory. The experimental period was 144 days. The number of eggs per gram (EPG) of feces was quantified on D21, D34, D48, D62, D76, D89, D103, D118, D131, and D144 post-infection. Pasture contamination with *H. contortus* L3 was examined. EPG in both groups of lambs was highest at D34. Egg shedding was significantly lower in both groups from D48 onwards, with a reduction of >95% from D103 onwards. Pasture contamination with L3 was highest at D41 but was then significantly lower in both groups. The total antioxidant capacity, the activity of glutathione peroxidase and the concentration of malondialdehyde in the serum changed significantly during the experiment (*p* < 0.003, < 0.001, and < 0.016, respectively). At least 54 species of meadow plants were identified on both pasture plots; plant bioactive compounds identified were mainly phenolic acids, flavonoids, and glucosides. Phenolic metabolites (e.g., coumaric acid, chicory acid, salvigenin, and esters of gallic acid) were identified in the feces of the lambs. In some lambs, the morphological observation identified small histopathological changes in the abomasal tissues typical of hemonchosis. Both the natural meadow pasture and the pasture enriched with experimentally sown chicory slowed the dynamics of GIN infection and pasture contamination with L3 by mobilizing the antioxidant defensive system and gradually increasing the resistance of the infected lambs, probably due to the beneficial effects of plant bioactive substances.

## 1 Introduction

Gastrointestinal parasites of ruminants represent a wide spectrum of genera and species; gastrointestinal nematodes (GINs) are of the greatest economic importance in Europe (1). Most herds/flocks in grass-based production systems are infected by GINs, and their main economic impact is due to sub-clinical infections that reduce animal growth and milk/wool production. *Haemonchus contortus*, a highly pathogenic nematode is considered the most important of GIN infections and is responsible for economic losses due to losses in productivity, high mortality, anthelmintic resistance, and cost of treatment (2–5). Antiparasitic drugs play a key role in combatting helminth and protozoan parasitic diseases in both production and pet animals worldwide (6). Effective anthelmintic therapy, however, has several risks and is considered unsustainable and non-ecological mainly due to the accumulation of drug residues in animal tissues and milk and to the development of resistance in parasitic nematodes to all commonly available anthelmintics (7–9). The goal of most methods for controlling parasites is not to eliminate the parasites, but to maintain the population at a level that does not affect the overall health of the host population (10).

The development of new preventive and therapeutic strategies against resistance to synthetic anthelmintics has increased the interest in plant medicines and other alternative methods of parasite control (11–13). Much work has focused on using plants with bioactive compounds in ruminants infected with GINs for their nutritional effects and anthelmintic activities (14–16). The diversity and synergy of bioactive compounds of several medicinal plants and their combinations, described in our previous studies (17, 18), contributed to some pharmacological efficacy against *H. contortus*. We therefore concluded that using treatments with bioactive plants against *H. contortus* could directly affect the dynamics of parasitic infection and indirectly mobilize the antioxidant defensive system and antibody or immune response in sheep, thus improving animal resistance (19, 20). Supplementation with only one medicinal bioactive plant (e.g., *Artemisia absinthium* or *Malva sylvestris*) also increased the resistance of lambs to *H. contortus* infection (21, 22). The antiparasitic properties of plant tannins and flavonoids were previously confirmed by several studies, indicating that including plants from the family *Fabaceae* (≥70% dry matter) in the feed could reduce the parasitic load in the abomasa of sheep infected with *H. contortus*, *Teladorsagia circumcincta*, and/or *Ostertagia ostertagi* and could decrease the number of eggs excreted in feces (23–25). GIN infections may also induce the production of reactive oxygen species, which may damage the parasites but generate oxidative stress (26).

Chicory (*Cichorium intybus*) is a perennial plant from the family *Asteraceae* widespread on the European continent that has been used as a feed additive for farm animals due to its high feed value and various therapeutic effects (27, 28). The anthelmintic effect of chicory and its secondary metabolites against GINs have been described in several studies (29–31). Chicory produces natural antioxidants that can be used in animal nutrition to protect from potentially harmful oxidative damage. We hypothesized that the grazing of sheep/lambs on a meadow with different plant species and on a meadow enriched with experimentally sown chicory may influence the level of lamb infection with GINs by reducing the number of parasite eggs in feces and adult worms in the abomasum and improving antioxidant status, which provides better defense against oxidative stress in infected lambs*. Haemonchus contortus* larvae developing within abomasal glands can cause major damage to the abomasal tissue, accompanied by the dilatation of, and damage to, the glands, hyperplasia in mucosal cells, and the loss of chief and parietal cells, so we were also concerned about the histopathological examination of the abomasa. Our goals were to investigate the effect of grazing on a natural meadow grassland enriched with experimentally sown chicory on (1) parasitological status, (2) pasture larval contamination, (3) antioxidative parameters in sera, and (4) the histopathology of the abomasa of lambs infected with *H. contortus*. We also qualitatively identified the main polyphenols in the meadow grassland and the phenolic metabolites in the feces.

## 2 Materials and methods

### 2.1 Animals, experimental plots, and design of the study

This study was conducted following the guidelines of the Declaration of Helsinki and National legislation in the Slovak Republic (G.R. 377/2012; Law 39/2007) for the care and use of research animals. The Ethical Committee of the Institute of Parasitology of the Slovak Academy of Sciences approved the experimental protocol on 20 January 2023. The experiment was carried out on pastures of a private farm (PETLAMB) in Petrovce, district of Prešov, Slovakia.

Two meadow grassland plots (0.43 ha each) with mixed plant species were fenced with electric fences; each plot had an automated water trough and a sheepfold. An area approximately 25% of the size of the experimental plot (CHIC) was recultivated with a mixture of three plants: 81.5% chicory (*Cichorium intybus*), 14.8% sainfoin (*Onobrychis viciifolia*), and 3.7% alfalfa (*Medicago sativa*). The control plot (CON) consisted exclusively of meadow grassland with mixed plant species. The plants of both plots (CON and CHIC) were identified based on plant atlas keys (32, 33). The land where these plots were established had never been grazed and was thus considered virtually worm-free.

The experiment was conducted between May and October. Sixteen male and female (1:1) Tsigai breed lambs aged 3–4 months with an average weight of 13.6 ± 0.52 kg from a sheep farm (PD Ružín–farm Ružín, Kysak, Slovakia) were transferred to the PETLAMB private farm. All lambs were dewormed with the recommended dose of albendazole (5 mg/kg body weight; Albendavet 1.9% suspension a.u.v, DIVASA-FARMAVIC S.A., Barcelona, Spain) during a 7-day period of adaptation before the start of the experiment. The animals were evenly divided into two groups of eight lambs based on their live weights and genders: the control group (CON) grazed the control plot, and the chicory group (CHIC) grazed the plot recultivated with chicory. Each animal was fed 300 g dry matter (DM) Mikrop ČOJ, a commercial concentrate (MIKROP, Čebín, Czech Republic) during the entire period of the study. Each lamb was experimentally infected with 5,000 third-stage larvae (L3) of the MHCo1 strain of *H. contortus* susceptible to anthelmintics (34); the day of infection was considered day 0 (D0) of the experiment. Both groups of lambs grazed the meadow grassland plots with mixed plant species for the first 33 days; the CHIC group started to graze the enriched pasture on D34. All animals were humanely slaughtered at the end of the trial (D144) following the rules of the European Commission (35) for slaughtering procedures.

### 2.2 Parasitological techniques

Fecal samples were collected rectally from each lamb on D21, D34, D48, D62, D76, D89, D103, D118, D131, and D144 post-infection. Part of these feces was stored at −80°C for subsequent analysis of polyphenols. The number of *H. contortus* eggs per gram (EPG) of feces was quantified using the modified McMaster method described by Coles et al. (36).

The percent fecal egg count reduction (%FECR) in the CON and CHIC groups was calculated using the formula (37):


$$\%\mathrm{FECR}=\left(1/n\right)\sum \left(100\times \left(1-\left[Ti_{2}/Ti_{1}\right]\right)\right)\text{,}$$

where *T*_*i*1_ is EPG on D34, and *T*_*i*2_ is EPG on the following sampling days (D48, D62, D76, D89, D103, D118, D131, and D144) in a host from a total of *n* hosts.

The humanely slaughtered animals were necropsied on D144. The gastrointestinal tract of each lamb was examined to count the total number of adult *H. contortus* in the abomasum. The abomasum of each animal was removed and dissected, and the contents were washed with warm physiological saline and emptied into a jar. The contents were mixed continuously to prevent the clustering of nematodes. Washings were brought up to a volume of two liters with physiological saline. Three 40-mL aliquots (5%) were collected from each animal and fixed with helminthological iodine. The number of adult *H. contortus* was counted for each animal.

To estimate pasture larval contamination and the survival of L3 over time, we collected grass samples from the experimental (CHIC) and control (CON) plots over 5 months on D0, D41, D78, D103, and D139. Up to 30 grass samples (5–10 g each) were cut from each plot 0–10 cm from the soil surface. We randomly collected grass samples on a “Z-shape trace” twice on each plot, cutting samples every 10–12 m. We also collected grass samples 0–20 cm from the edge of sheep feces in the area around the sheepfold. Samples from the CON and CHIC plots were collected and examined separately.

Infective larvae of *H. contortus* were collected from the grass samples using the Baermann procedure. Twenty grams of grass subsamples were cut manually by scissors and placed in a Baermann funnel with tap water for 10–12 h (overnight); we used 6 to 12 Baermann funnels for each CON and CHIC plot. L3 were collected from the bottom of the funnel, stained with iodine to separate the L3 from free-living soil nematodes, and counted under a microscope with 40× magnification.

The pasture larval contamination was expressed as the number of L3 per kilogram of dry grass matter (L3/kg DM) using the formula:


$$L3/\mathrm{kg}\;DM=50\times n_{av}\text{,}$$

where n*_av_* is the average number of L3 in 20 g of grass subsample.

### 2.3 Antioxidant parameters

Blood samples for sera were collected from each animal on D0, D21, D34, D62, D89, and D131 and placed directly into 10-mL serum separator tubes (Sarstedt AG & Co, Nümbrecht, Germany) on the farm and delivered to the laboratory. The samples were centrifuged at 1200 *g* for 10 min at room temperature to separate the serum; the sera samples were stored at −80°C until analysis. The total antioxidant capacity (TAC), the activity of glutathione peroxidase (GPx), and the concentration of malondialdehyde (MDA) in the sera were determined as previously described (38).

### 2.4 Analysis of polyphenols

Samples of plant species from the meadow grassland plots were collected from 10 random quadrats (0.2 × 0.2 m each) of land grazed by the CON and CHIC groups from May to September. The polyphenols were analyzed as previously described (28).

The frozen feces were freeze-dried using a Gamma 2–16 LSC freeze dryer (Christ, Osterode, Germany), ground with a ZM 200 mill (Retsch, Düsseldorf, Germany) equipped with a 1 mm sieve, and stored in the dark for subsequent use and analysis. Three random samples, each 100 mg, were extracted three times with 80% MeOH for 30 min at 40°C (ultrasonic bath). The extract was evaporated to dryness and then dissolved in 2 mL of Milli-Q water (acidified with 0.2% formic acid) and purified by Solid Phase Extraction using an Oasis HLB 3 cc Vac Cartridge, 60 mg (Waters Corp., Milford, USA). The cartridge was washed with 0.5% methanol to remove carbohydrates and then washed with 80% methanol to elute phenolics, then re-evaporated to dryness and dissolved in 1 mL of 80% methanol (acidified with 0.2% formic acid). The sample was then centrifuged (23,000 *g*, 5 min) before spectrometric analysis. All analyses were performed in triplicate for three independent samples and stored at −20°C before analysis. Polyphenol composition was estimated using liquid chromatography electrospray ionization (ESI) quadrupole time-of-flight (QTOF) mass spectrometry on a Thermo Dionex Ultimate 3,000 RS chromatographic system (Thermo Fisher Scientific, Waltham, USA) coupled to a Bruker Compact QTOF mass spectrometer (Bruker, Billerica, USA), consisting of a binary pump system, sample manager, column manager, and photodiode array detector. Separations were performed on a 2.1 × 100 mm, 2.6 μm Kinetex C18 column (Phenomenex, Torrance, USA), with mobile phase A consisting of 0.1% (v/v) formic acid in water and mobile phase B consisting of 0.1% (v/v) formic acid in acetonitrile. A linear gradient from 5 to 60% phase B in phase A over 20 min was used to separate phenolic compounds. The flow rate was 0.3 mL/min, and the column was held at 30°C. Spectra were acquired in negative-ion mode over a mass range from *m/z* 100 to 1,500 with a frequency of 5 Hz. The operating parameters of the ESI ion source were: capillary voltage, 3 kV; dry-gas flow, 6 L/min; dry-gas temperature, 200°C; nebulizer pressure, 0.7 bar; collision radio frequency, 700.0 V; transfer time, 100.0 μs; and pre-pulse storage, 7.0 μs. Ultrapure nitrogen was used as the drying and nebulizer gasses, and argon was used as the collision gas. The collision energy was set automatically from 15 to 75 eV depending on the m/z of the fragmented ion. The data acquired were calibrated internally, with sodium formate introduced to the ion source at the beginning of each separation via a 20-μL loop.

The spectra were processed using Bruker DataAnalysis 4.3 software (Bruker Daltonics GmbH, Bremen, Germany), which provides a ranking based on the best fit of measured and theoretical isotopic patterns within a specific window of mass accuracy. The quality of the isotopic fit was expressed using the mSigma-value. SmartFormula3D matched peaks were sent to the MetFrag website *in silico* fragmentation for the computer-assisted identification of metabolite mass spectra. We also used a few databases to search for the structural identity of the metabolites: the human metabolome database,^1^ the BiGG database,^2^ the PubChem database,^3^ the MassBank database,^4^ KEGG,^5^ and the Metlin database,^6^ supported with appropriate literature information (39–42).

### 2.5 Histological examination

Abomasal tissues were histologically examined similarly to the procedure previously described (43). Samples of fresh tissues from the abomasum of each lamb were washed in a phosphate buffer (0.1 M, pH 7.4), put in plastic containers, and fixed in a 10% buffered formalin solution as pieces of tissue spread on flat polystyrene. The fixed material was processed using a series of reagents and embedded in Paraplast PLUS paraffin blocks (Leica, Buffalo Grove, USA), which were then cut with a rotary microtome into sections 3.5 μm thick. Slides with a paraffin section were automatically stained with hematoxylin and eosin (Varistain Gemini Thermo Scientific, Runcorn, UK). An Axio Lab. A1 microscope (Carl Zeiss, Jena, Germany) equipped with a Zeiss Axiocam ERc5s digital camera was used for the histological examination. Photographs were analyzed and recorded using ZEN 2.3 (blue edition) software (Carl Zeiss Microscopy GmbH, 2011).

### 2.6 Statistical analyses

The data were statistically analyzed using GraphPad Prism (9.2.0 (332) 2021; GraphPad Software, Inc., San Diego, USA). Parasitological data were analyzed using an unpaired *t-*test. Data for the antioxidant parameters of the blood sera were analyzed using two-way analyses of variance. The model included treatment, time, and the interaction between treatment and time. Results were considered significant at *p* < 0.05.

## 3 Results

### 3.1 Parasitological status

**Figure 1:**
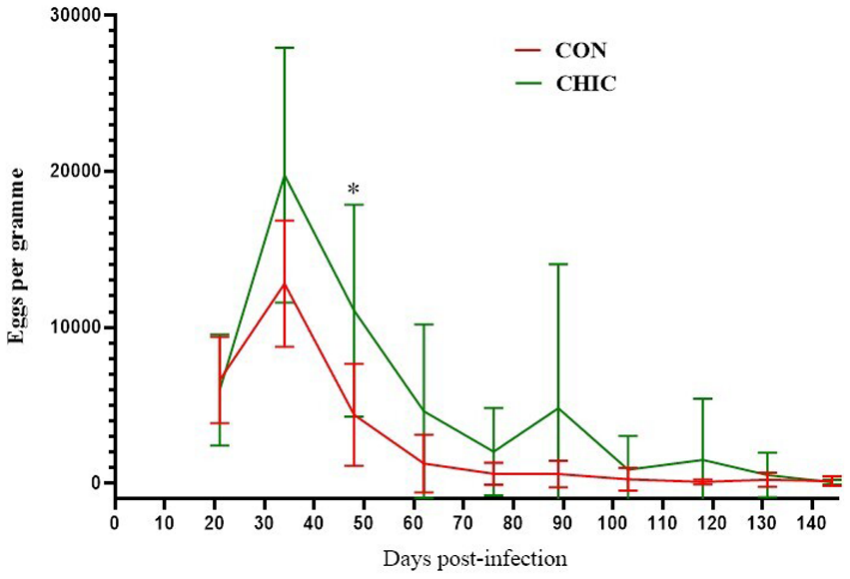
Mean fecal egg counts ± SD for the control (CON) and chicory (CHIC) groups of lambs infected with *Haemonchus contortus.*

We first noticed a reduction in egg shedding in the feces of both groups of lambs on D48 (Figure 1). %FECR confirmed reductions of 63.94% (*p* < 0.004) and 40.58% (*p* < 0.037) of egg number in the CON and CHIC groups, respectively (Table 1). The egg shedding reductions (94.48–99.77%) were highest from D103 onwards in both groups. The mean number of abomasal worms recorded per lamb after the necropsy was lower in the CON (59.33) than the CHIC (88.63) group, but these data were not significant (*p* > 0.05).

**Table 1:** Percent fecal egg count reduction (%FECR) of the control (CON) and chicory (CHIC) groups of lambs (*n* = 8).

	%FECR in days post-infection							
	D48	D62	D76	D89	D103	D118	D131	D144
CON	63.94	86.0	94.96	93.04	97.16	99.6	97.9	98.53
*p*	0.004	< 0.001	< 0.001	< 0.001	< 0.001	< 0.001	< 0.001	< 0.001
CHIC	40.58	77.66	91.46	83.0	96.85	94.48	98.06	99.77
*p*	0.037	0.007	< 0.001	0.004	< 0.001	< 0.001	< 0.001	< 0.001

%FECR, expressed as a percentage of egg reduction to day 34 post-infection.

The pasture larval count was highest at D41 in both groups of lambs (Table 2); the number of L3 on “random” grass was 20.9-fold higher on the CON (2,820 L3/kg DM) than the CHIC (135 L3/kg) plot. The number of *H. contortus* larvae on the grass samples collected near feces at D41 was similar on both plots. Subsequent sampling identified significantly reduced pasture larval contamination on both plots and on D139 larvae were not found on the grass of either plot. *Haemonchus contortus* was the only nematode species found in the pasture grass samples.

**Table 2:** Pasture larval contamination expressed as the number of infective third-stage larvae (L3) per kilogram of dry grass matter (L3/kg DM) in the control (CON) and chicory (CHIC) groups of lambs.

Grass sample	Day	Group of lambs	
		CON	CHIC
Random grass sample	D0	0	0
	D41	2,820	135
	D78	87.5	12.5
	D103	29	12.5
	D139	0	0
Grass near **feces**	D0	0	0
	D41	11,900	11,120
	D78	10	0
	D103	83	50
	D139	0	0

### 3.2 Antioxidant status

TAC, lipid peroxidation (i.e., MDA concentration), and GPx activity in the blood serum changed significantly during the experiment (*p* < 0.003, < 0.001, and < 0.016, respectively) (Table 3). GPx activity continuously decreased throughout the experiment in both the CON and CHIC groups. TAC was lower on D21 and D34, and lipid peroxidation was higher on D21, D34, and D62, in both the CON and CHIC groups. TAC and MDA concentrations on D131 were similar to their initial values at the beginning of the experiment (D0) for both groups.

**Table 3:** Antioxidant parameters in the blood sera of the control (CON) and chicory (CHIC) groups of lambs (*n* = 8).

Parameter	Day	CON	CHIC	SD	Treatment (Tr)	Time (T)	Tr × T
TAC (mmol/L)	D0	0.507	0.500	0.115	0.907	0.003	0.230
	D21	0.411	0.471	0.043			
	D34	0.457	0.486	0.051			
	D62	0.518	0.457	0.066			
	D89	0.539	0.540	0.064			
	D131	0.523	0.509	0.056			
GPx (U/mL)	D0	0.113	0.105	0.040	0.319	<0.001	0.563
	D21	0.092	0.108	0.032			
	D34	0.065	0.063	0.037			
	D62	0.062	0.064	0.029			
	D89	0.054	0.054	0.015			
	D131	0.050	0.079	0.028			
MDA (μmol/L)	D0	0.195	0.184	0.032	0.072	0.016	0.412
	D21	0.226	0.229	0.046			
	D34	0.221	0.226	0.032			
	D62	0.250	0.216	0.055			
	D89	0.240	0.186	0.062			
	D131	0.192	0.183	0.053			

TAC, total antioxidant capacity; GPx, activity of glutathione peroxidase; MDA, concentration of malondialdehyde.

### 3.3 Grassland polyphenols and phenolic metabolites in feces

The diversity of the meadow plant species in the samples collected from the plots grazed by the CON and CHIC groups from May to September was high; at least 54 species from 20 families were collected and identified (Table 4). The analyses of plant bioactive compounds identified mainly phenolic acids, flavonoids, and glucosides in the samples from the CON and CHIC plots.

**Table 4:** Plant species and main polyphenolic compounds in the pasture plots from May to September.

Family and species	Polyphenolic compounds
Apiaceae *Astrantia major*, *A. carniolica*, *Daucus carota*; Asteraceae *Achillea millefolium*, *A. nobilis*, *Centaurea nigrescens*, *Cichorium intybus*, *Cirsium oleraceum*, *C. vulgare*, *Crepis sancta*, *Erigeron annuus*, *Hypochaeris glabra*, *Chamaemelum nobile*, *Solidago nemoralis*, *Tussilago farfara*; Brassicaceae *Lepidium draba*; Campanulaceae *Campanula patula*; Caprifoliaceae *Knautia arvensis*; Convolvulaceae *Calystegia sepium*; Cyperaceae *Carex hirta*; Dryopteridaceae *Dryopteris filix-mas*; Equisetaceae *Equisetum sylvaticum*; Fabaceae *Trifolium pratense*, *T. repens*, *Vicia hirsuta*, *V. sepium*; Gentianaceae *Centaurium erythraea*; Geraniaceae *Geranium maculatum*, *G. pratense*; Hypericaceae *Hypericum perforatum*; Lamiaceae *Clinopodium vulgare*, *Galeopsis pubescens*, *Mentha longifolia*, *Prunella vulgaris*, *Stachys officinalis*, *S. palustris*, *Thymus pulegioides*; Lythraceae *Lythrum salicaria*; Plantaginaceae *Plantago lanceolata*, *P. major*, *Veronica spicata*; Poaceae *Agrostis capillaris*, *Calamagrostis arundinacea*, *C. epigejos*, *Setaria pumila*; Primulaceae *Lysimachia vulgaris*; Rosaceae *Alchemilla xanthochlora*, *Filipendula ulmaria*, *F. vulgaris*, *Fragaria vesca*, *Prunus domestica*, *Rosa canina*, *Rubus ursinus*; Urticaceae *Urtica dioica*	trans 3-O-Caffeoylquinic acid, 5-O-p-Coumaroylquinic acid, trans 4-O-Caffeoylquinic acid, trans 5-O-Caffeoylquinic acid, deriv. Caffeoylquinic acid, O-p-Coumaroylquinic acid, D-chicoric acid, Pleoside, Plantamajoside; Luteolin 7-O-diglucuronide, Neocarlinoside, Isocarlinoside, Isovitexin 2”-O-arabinoside(Vitexin 2″-xyloside), Orientin, Homoplantaginin, Quercetin 3,7-dirhamnoside, Quercetin 3-neohesperidoside, rhamnosyl-glucoside, Luteolin C-glucoside C-xyloside, Schaftoside, Apigenin 6-C-glucoside(−6”-O-arabinoside), Vicenin II, Protocatechuic acid 4-glucoside, Vanillic acid 4-beta-D-glucoside, Pyrogallol-2-O-glucuronide, Trihydroxy butanoic glucoside, Vitexin xyloside, Isolariciresinol glucuronide, Acteoside, Isoacteoside, Tricin rutinose, Geniposidic acid, Decaffeoylverbascoside, 8-Epiloganic acid, Isovitexin 6”-O-glucoside, Quercetin 3-neohesperidoside, Hesperetin 7-(2,6-dirhamnosylglucoside), Acteoside, Malvidin, Tricin 7-neohesperidoside, L-Tryptophan, 4-Hydroxybenzoyl glucose, Azelaic acid, Lavandulifolioside. Malic acid, Quinic acid, cis-Caftaric acid, Cichorioside, (−)-esculin, 3-O-Caffeoylquinic acid, 5-O-Caffeoylquinic acid, Caffeic acid, trans 4-O-Caffeoylquinic acid, Quercetin 3,5-diglucoside, 5-p-Coumaroylquinic acid, Ascochitine, Cyanidin 3-glucogalactoside, Feruloylquinate, Cyanidin 3-gentiobioside, Cyanidin 3-galactoside, Chicoric acid, D-chicoric acid, Quercetin 3-rutinoside, Quercetin-4′-glucuronide, Kaempferol 3-O-glucuronide, Quercetin 3-(6″-malonyl-glucoside), 3,4-Dicaffeoylquinic acid, Kaempferol 7-O-glucoside, Isorhamnetin 7-glucuronide, 3,5-Dicaffeoylquinic acid, Quercetin 3-(6″-acetylglucoside), Kaempferol 3-O-(6-malonyl-glucoside), Intybin

**Figure 2:**
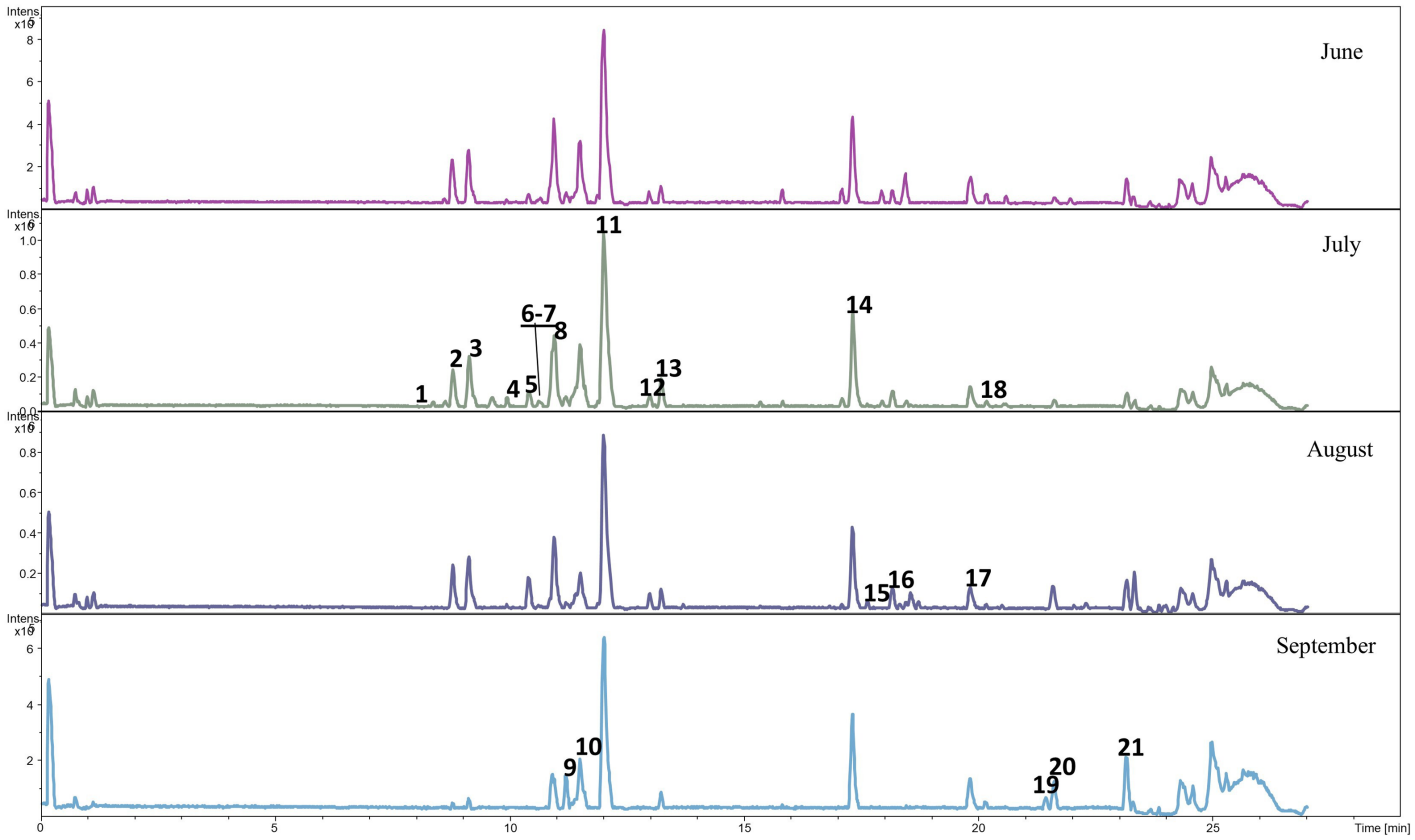
Base peak chromatogram of phenolic metabolites in the feces of lambs from the control group. The peak numbers represent the main phenolic metabolites as follows: **1** cumarinic acid, **2** tridec-3-enedioic acid, **3** D-chicoric acid, **4** dihydrophaseic acid, **5** 3′,4′,5′-trimethoxy flavonol, **6** azaleic acid, **7** salvigenin, **8** 2′,3-dihydroxy-4,4′,6′-trimethoxychalcone, **9** 7-hydroxy-2′,3′,4′-trimethoxyisoflavan, **10** 2′,3-dihydroxy-4,4′,6′-trimethoxychalcone (C), **11** 2′,3-dihydroxy-4,4′,6′-trimethoxychalcone (diapocynin), **12** 4-hydroxyequol, **13** uralenneoside, **14** 9-hydroxy-hexadecane-1,16-dioate, **15** dodecanedioic acid, **16** epi-lipoxin, **17** 2,3-dihydroxy-5-undecylcyclohexa-2,5-diene-1,4-dione, **18** tetradecanedioic acid, **19** 3,7-dihydroxycholan-24-oic acid, **20** 7alpha-hydroxy-3-oxo-5beta-cholan-24-oic acid, and **21** isodeoxycholic acid.

**Figure 3:**
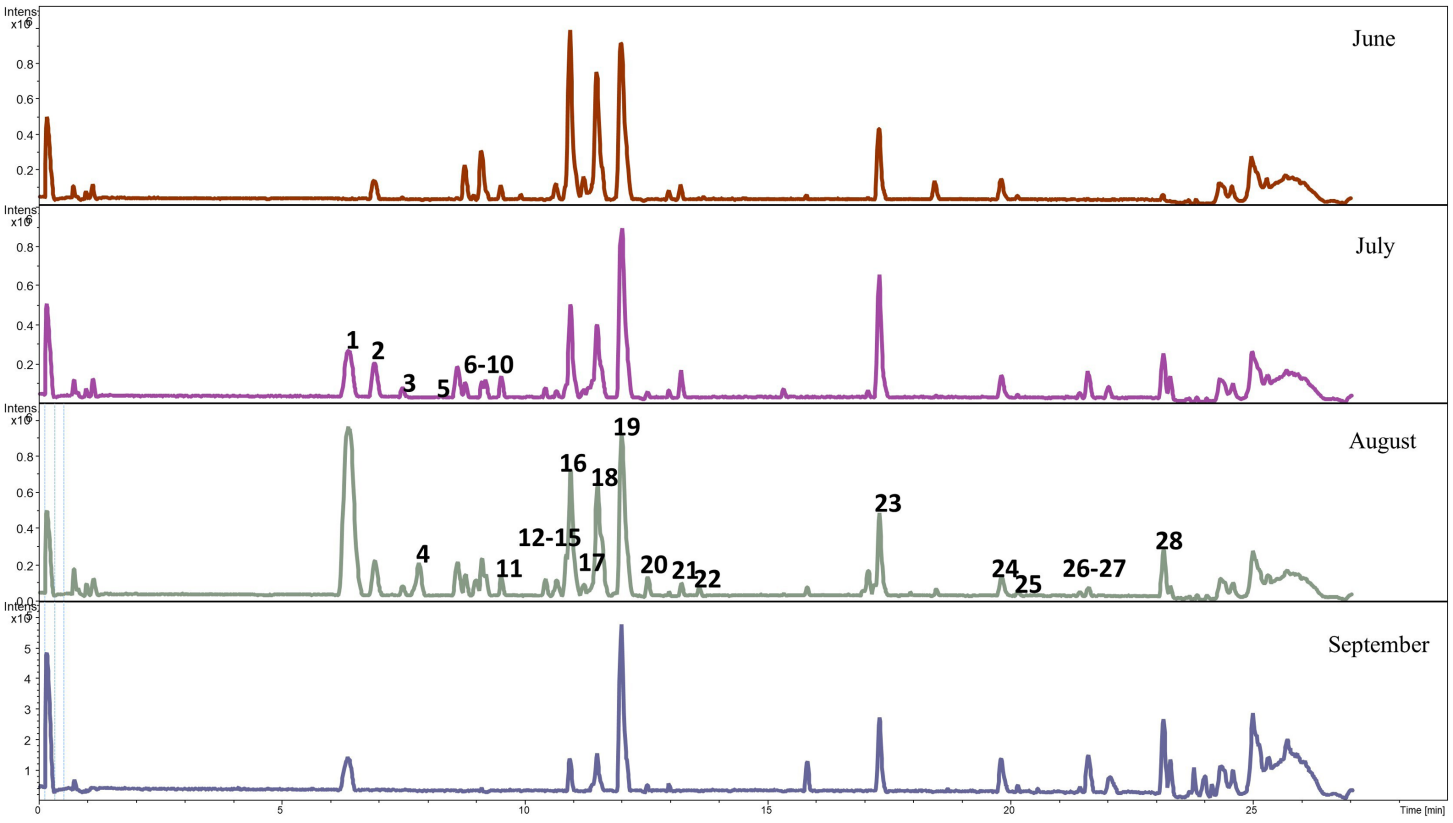
Base peak chromatogram of phenolic metabolites in the feces of lambs from the chicory group. The peak numbers represent the main phenolic metabolites as follows: **1** Not determined, **2** gallic acid octyl ester, **3** gallic acid octyl ester, **4** ND, **5** cumarinic acid, **6** (S)-2,3-dihydro-7-hydroxy-2-methyl-4-oxo-4H-1-benzopyran-5-acetic acid, **7** ND, **8** 3-methoxyphenylacetic acid, **9** tridec-3-enedioic acid, **10** pentadeca-4,7,10-trienedioic acid, **11** D-chicoric acid, **12** ND, **13** 2′,7-dihydroxy-4′,6-dimethoxyisoflavan, **14** 3′,4′,5′-trimethoxyflavonol, **15** salvigenin, **16** 2′,3-dihydroxy-4,4′,6′-trimethoxychalcone, **17** 7-hydroxy-2′,3′,4′-trimethoxyisoflavan, **18** 2′,3-dihydroxy-4,4′,6′-trimethoxychalcone (melilotocarpan C), **19** 2′,3-dihydroxy-4,4′,6′-trimethoxychalcone (diapocynin), **20** dihydrophaseic acid, **21** 4-hydroxyequol, **22** uralenneoside, **23** dodecanedioic acid, **24** 2,3-dihydroxy-5-undecylcyclohexa-2,5-diene-1,4-dione, **25** tetradecanedioic acid, **26** 3,7-dihydroxycholan-24-oic acid, **27** 7alpha-hydroxy-3-oxo-5beta-cholan-24-oic acid, and **28** isodeoxycholic acid.

Qualitative analyses of phenolic metabolites in the feces of the lambs from the CON and CHIC groups identified 21 and 28 phenolic metabolites, respectively (Figures 2, 3). The peak numbers in Figures 2, 3 represent the list of the main phenolic metabolites in the feces of the lambs from both groups.

### 3.4 Histology of abomasal tissue

**Figure 4:**
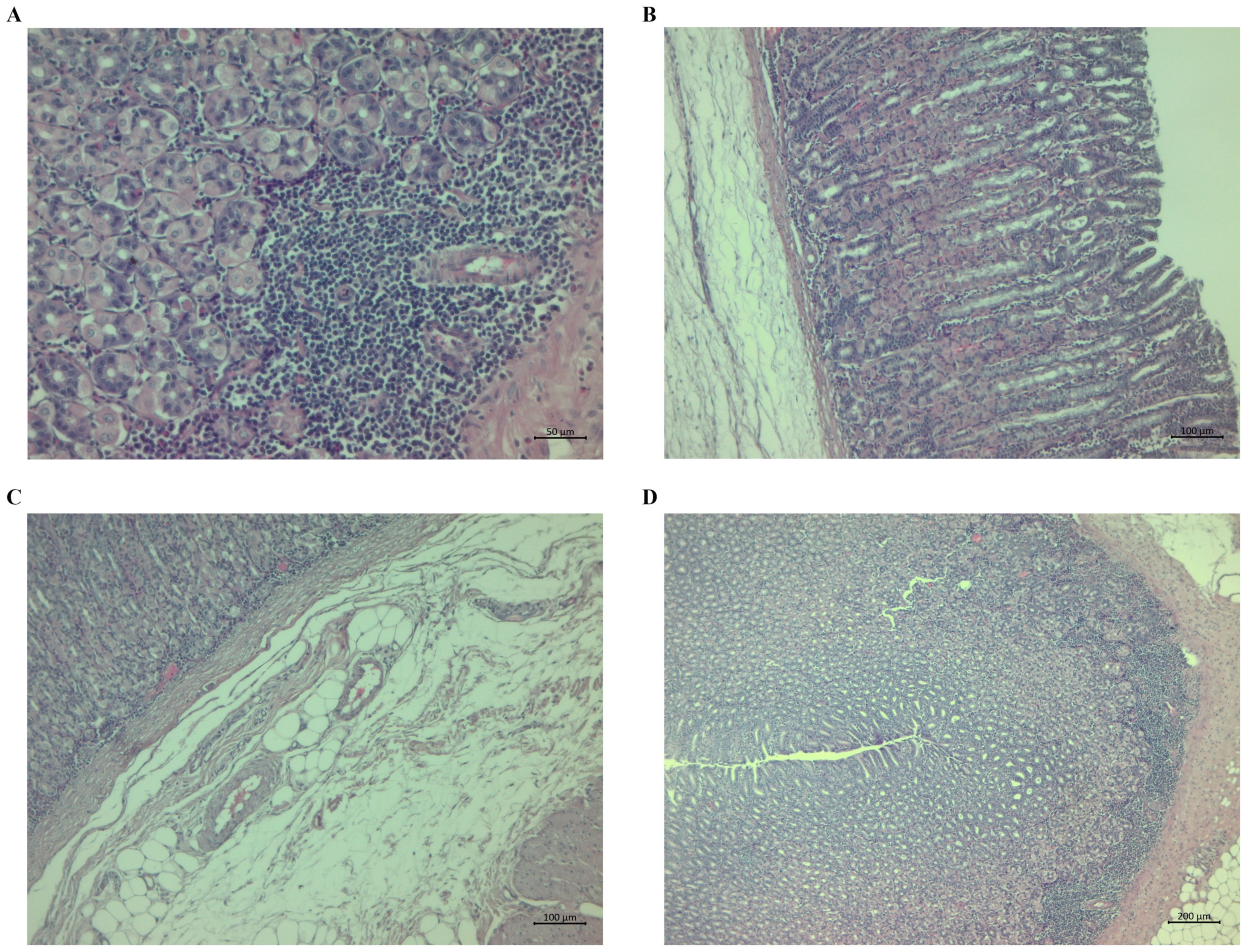
Histopathological sections of abomasa from the control group infected with *Haemonchus contortus*. **(A)** Sections stained with hematoxylin and eosin (H&E) (200×) showing aggregates near and in the *tunica muscularis* and gastric mucosa. **(B)** Sections stained with H&E (100×) showing submucosal oedema with scattered lymphocytes and dilatation of glands. **(C)** Sections stained with H&E (100×) showing severe submucosal oedema and the presence of lymphocytes near the *t. muscularis*. **(D)** Sections stained with H&E (40×) showing mucosal hypertrophy, marked lymphocyte aggregates, and infiltration.

Morphological observation identified histological changes in the abomasal tissues of the lambs typical for haemonchosis. The changes in the CON group were aggregates near and in the *tunica muscularis* and gastric mucosa (Figure 4A) and submucosal oedema with scattered lymphocytes and glandular dilation (Figure 4B). We also observed submucosal oedema and the presence of lymphocytes near the *t. muscularis* in some cases (Figure 4C). Some animals in the CON group had mucosal hypertrophy and prominent lymphocyte aggregates with infiltration (Figure 4D).

**Figure 5:**
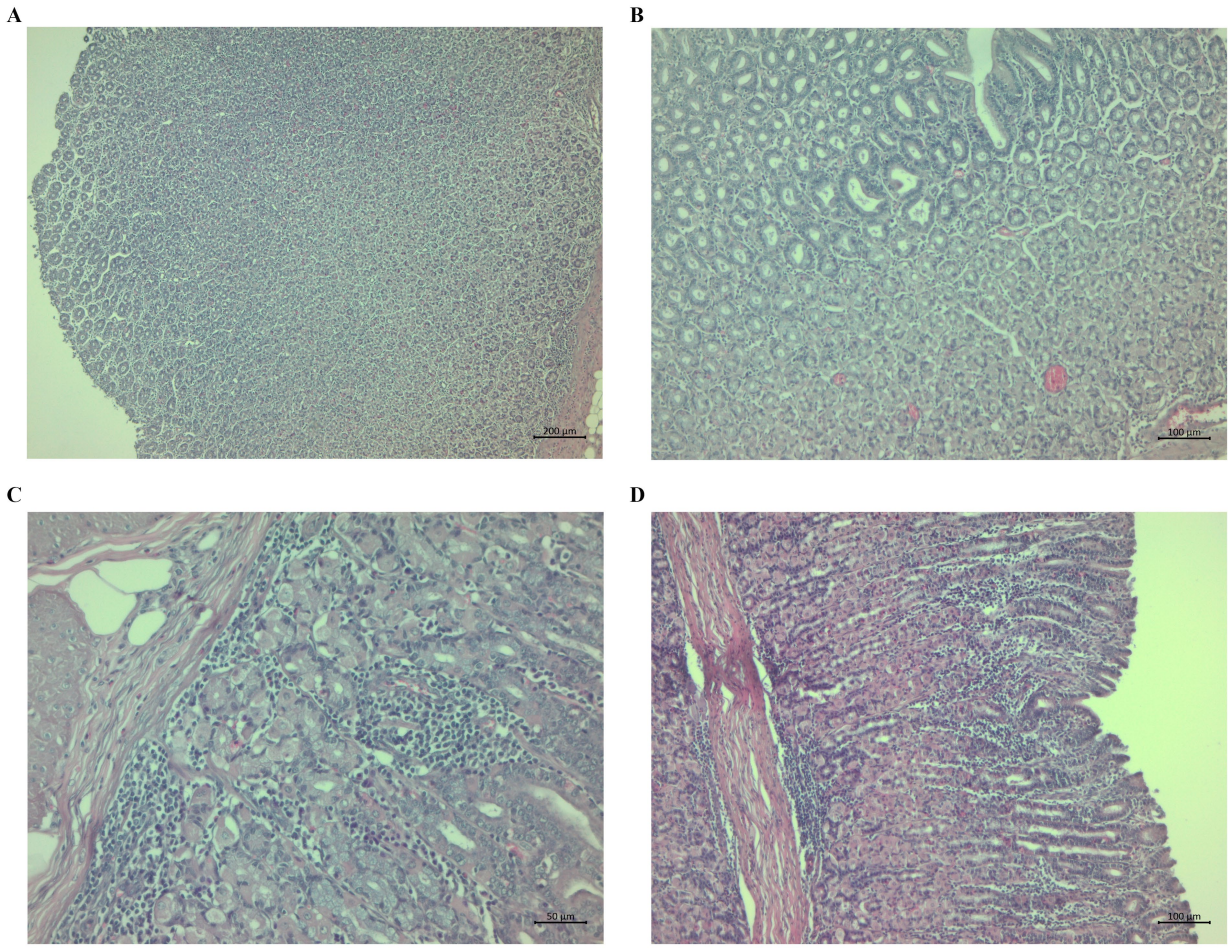
Histopathological sections of abomasa from the chicory group infected with *Haemonchus contortus*. **(A)** Sections stained with hematoxylin and eosin (H&E) (40×) showing hypertrophy of the mucosa. **(B)** Sections stained with H&E (100×) showing hypertrophy of the mucosa, mild hyperaemia, and dilatation of glands. **(C)** Sections stained with H&E (200×) showing aggregates near the *tunica muscularis* and gastric mucosa and scattered throughout the gastric mucosa. **(D)** Sections stained with H&E (100×) showing lymphocyte aggregates and infiltration.

The changes in the CHIC group were hypertrophy of the mucosa (Figure 5A) and mild hyperaemia and dilatation of glands (Figure 5B). Other pathological changes in the abomasal tissue included aggregates near the *t. muscularis* or scattered throughout the gastric mucosa (Figure 5C) and lymphocytic aggregates and infiltration (Figure 5D).

## 4 Discussion

### 4.1 Parasitological status

We hypothesized that the natural meadow grassland enriched with experimentally sown chicory would influence the infection of lambs with *H. contortus*. We initially expected that egg excretion would gradually decrease in the experimental CHIC group throughout the experiment. The results of this study, however, clearly indicated that the fecal shedding of eggs also decreased significantly (*p* < 0.05) in the CON group from D48 onwards. This decrease was even larger up to D89 than in the CHIC group. The reduced egg production was probably correlated with the reduction in the number of adult worms in the abomasa as a direct correlation between EPG value and the number of GINs recorded for *H. contortus* in sheep and other ruminants (44).

Different nutritional strategies for managing GINs and *H. contortus* in ruminants (45–47) have already been examined, but comprehensive recommendations and mechanisms of action are still not unified. Chicory meets the criteria of a suitable antiparasitic plant due to *in vitro* and *in vivo* evidence of its anthelmintic effects, is voluntarily grazed by ruminants, can be cultivated as fodder, and is widespread in middle climatic zones (48). *In vitro* anthelmintic activity of chicory leaves and roots was confirmed by the inhibition of the hatching of Trichostrongylidae eggs with significant efficacies of 62.6% and higher (49). In a study on the effect of chicory compared to meadow hay in sheep infected with mixed Trichostrongylidae infections (50), a progressive increase in egg shedding by 80.83% on day 28 was observed in the group of sheep fed with meadow hay. Continuous reduction (by 24.75% after 28 days) in parasitic load in the chicory hay group was recorded (50). They observed a higher reduction (by 77.6%) after grazing on chicory for 28 days (at D62). The reduction in EPG value in the CHIC group, however, cannot be attributed to the action of chicory alone, because the reduction from D48 to D89 was 3.5–23.4% higher in the CON group. Heckendorn et al. (24) reported a similar course of infection with *H. contortus* in lambs administered with chicory, where the fecal egg counts peaked at the beginning of the study and then decreased significantly by 69%, confirming that tanniferous forages such as chicory were associated with significant reductions in the total daily fecal egg output of *H. contortus*. The peak of infection in the control group in the present study was not as high as in the lambs from the chicory group, similar to our experiment; thus, Heckendorn et al. (24) assumed that chicory had a direct antiparasitic effect. Our results indicated that infection in both groups was highest on D34, but the mean EPG was lower in the CON than the CHIC group, which was able to respond to the antiparasitic properties of the meadow grassland in the CON pasture. Our previous studies have described the antiparasitic properties of some plants in meadow grasslands grazed by CON lambs (17, 18, 34, 51).

The pathogenic effect of the GINs of ruminants also increases with the rate of stocking (52). The supplementation of lambs with food concentrates and the availability of proteins to replace lost proteins caused by parasitic infection also play a very important role (53). The combination of commercial food concentrate with the anthelmintic efficacy of some plants in our study (Table 4) could probably account for the significant decrease in excretion of *H. contortus* eggs in the CON group. EPG on D144 was lower in the CHIC than the CON group (Figure 1), which did not fully correlate with the necropsy results, where the total number of adult worms in the abomasum was higher in the CHIC group. The number of adult worms and the difference between the CHIC and CON groups, however, were not statistically significant. These findings suggest that worms from the CHIC group could be less fertile after exposure to chicory than worms from the CON group. The fecundity of female *T. circumcincta* exposed to chicory in another study did not differ from the control group (54), but similar data for *H. contortus* and this phenomenon are not available.

The positive antiparasitic effect of feeding or grazing experimentally infected calves on forage chicory, represented by a significant reduction in parasitic load and EPG value for *O. ostertagi*, has also been demonstrated (55). The welfare of grazing ruminants could be improved by eliminating negative human-animal interactions, such as the use of pharmacological tools (56). Herbivores can cope with parasitic infections by foraging, which includes mechanisms for preventing infection, resisting parasitism, and self-medicating by eating plants with anti-parasitic properties (57). Infected lambs grazing on rich grasslands are probably able to seek out plants with specific medicinal, antioxidant, immunological (58, 59), and anthelminthic effects and probably increase their consumption when self-medicating against GINs (60). Some polyphenols have synergistic effects. Significant synergies against parasitic infections in sheep have been reported for a combination of medicinal plants containing alkaloids, condensed tannins, flavonoids, and proteases, suggesting complex biochemical interactions that have strong anthelmintic effects (61). These factors may have played a critical role in reducing parasitic infection in both groups of lambs in our experimental study.

Our study of changes in the level of pasture contamination with *H. contortus* L3 provided interesting results. The level of contamination with L3 on both pasture plots increased in the first stage of the experiment (until D41), which was associated with the peculiarities of the development of *H. contortus* in the environment (62, 63). L3 contamination of the “random” pasture grass, however, was 20.9-fold higher on the CON than the CHIC plot, despite the similarity of the EPG values in the CON and CHIC lambs during this period and the similar high contamination of grass “near the feces” around the sheepfolds (Table 2). This difference in the contamination of the pasture plots may have been due to the lower survival of L3 on the plot recultivated with chicory compared to the natural meadow grass. *Haemonchus contortus* L3 survives and migrates differently in pastures with different types of vegetation, probably due to the peculiarities of the composition of the vegetation species and the structure of the meadow plant species (64–67). The physiological and morphological peculiarities of chicory may hinder larval survival on pasture. We also believe that several bioactive compounds and phenolic metabolites in lamb feces also influence the successful development of eggs and free-living larvae in the feces. The number of phenolic metabolites in our study was higher in the feces of the CHIC than in the CON lambs (Figures 2, 3). Chicory and its extracts negatively affect the development of eggs and larvae of various ruminants (54, 68, 69), so we believe that the decrease in the level of pasture contamination with L3 in our study was associated with the influence of bioactive plants, mainly chicory.

The subsequent dramatic reduction of pasture contamination with *H. contortus* larvae (at D78 and later) was consistent with the results of the FECR test. The lambs were not treated with any anthelmintics during this period, so we believe that the reduction in EPG values in the lambs and the level of L3 contamination in the pastures were associated with the influence of diverse phenolic metabolites on eggs and free-living larvae in the lamb’s feces. Approximately 95% of the parasite population in pastures is presented as eggs and free-living larvae, with only 5% of the population present as adults in animals (70), so the direct effect of bioactive plants and phenolic metabolites on the survival of eggs and larvae on pasture is a very important factor for GIN control. We therefore suggest that using bioactive plants to improve pasture management and reduce L3 intake in grazing ruminants may be a promising approach to control parasites in sheep and other ruminants.

### 4.2 Antioxidant status

The results of our experiment indicated that both the CON and CHIC pastures containing bioactive compounds had strong antioxidant properties, with a beneficial effect on the antioxidant status of infected lambs. The polyphenols in pasture grass likely provided natural antioxidants to minimize oxidative stress in the lambs, although antioxidant potential may have been influenced by several factors, including intestinal absorption, metabolism, bioavailability, and the presence or absence of co-antioxidants and transition metal ions (71). These findings are consistent with our previous results of using medicinal plants with a wide range of bioactive compounds that had strong antioxidant potential in the diet of lambs infected with *H. contortus* (18, 19, 34, 38, 51). Similarly, the plants in the grasslands of both the CON and CHIC pastures were good sources of flavonoids and phenols, which have antioxidant activity. Increasing the intake of medicinal plants on pasture likely helped to maintain the proper level of antioxidant potential and reduced the risk of infection associated with oxidative stress (72, 73). The antioxidant status of the infected lambs, however, probably also depended strongly on the efficiencies of the absorption, concentration, and metabolic transformation of phytochemicals and the environment (74).

### 4.3 Meadow grassland polyphenols and phenolic metabolites in feces

To the best of our knowledge, this study is the first to perform a large-scale analysis of bioactive compounds on both pasture and the feces of lambs burdened with endoparasites. The natural meadow grassland with a diversity of plant species and enriched with experimentally sown chicory in our experiment was a source of important bioactive compounds that acted synergistically, with important biological effects (e.g., antioxidant, anti-inflammatory, or anthelmintic) in the treatment of GIN infection (75–77). Flavonoids (e.g., quercetin, luteolin, and kaempferol), flavonoid glycosides, and many flavonoid derivatives were the most abundant bioactive compounds identified in the pastures grazed by the CON and CHIC lambs, so they likely influenced GIN infection both directly and indirectly. Flavonoid glycosides and tannins have a similar mechanism of direct action against GINs due to their similar chemical structures (78) and have synergistic anthelmintic effects when tannins are combined with quercetin or luteolin (79). Flavonoids, however, are also known for their indirect activity, by which they increase host resistance to GINs (80, 81) by mobilizing the antioxidant defensive system, allowing animals to protect themselves from new oxidative conditions, as in our experiment. Finally, the phenolic acids, quinic acid, caffeic acid, and coumaric acid derivatives, which also have antioxidant, anti-inflammatory, antibacterial, and anthelmintic properties (82–84), were identified in the plots grazed by the CON and CHIC lambs.

The bioavailability, absorption, and distribution of polyphenols from the plots grazed by the CON and CHIC lambs were likely influenced by direct interactions between polyphenols and feed components in different parts of the digestive tract (85, 86). Some ingested flavonoids may be broken down into phenolic acids, but 90–95% of all ingested polyphenols may accumulate in the colonic lumen, and phenolic metabolites produced by microbes may be absorbed or excreted in the feces (87). The gut microbiota in both the CON and CHIC lambs was likely responsible for the extensive degradation of the original polyphenolic structures to a series of low-molecular-weight molecules that were absorbed and thus responsible for the biological activity. In the feces of both groups, we identified coumaric acid, a phenolic compound with a broad spectrum of biological activities (i.e., antimicrobial, antioxidant, and anti-inflammatory) (88), or chicoric acid, which has anti-inflammatory and antioxidant effects in digestive diseases (89). The flavonoid salvigenin, typical for *Salvia* species, has a wide range of biological and pharmacological properties and was abundantly represented in the feces of the lambs from both the CON and CHIC groups (90). These identifications of bioactive compounds in the feces were of great importance for the identification of the active phenolic metabolites in particular, because they likely had a specific effect against parasitic GIN larvae on pasture. The composition of polyphenols clearly varies considerably depending on the type of polyphenols and the feed source, but monomeric forms of flavonoids, for example, are best protected from degradation in ruminants, and their bioavailability is high (91). Our results confirmed that lambs can learn to prefer some bioactive plants during therapeutic self-medication or may adjust their intake when sick during nutritional self-medication, but the sustained consumption of bioactive plants can clearly lead to animals being tolerant to disease during prophylactic self-medication (92).

### 4.4 Histopathology

Morphological observation identified histopathological changes in the abomasal tissues typical of haemonchosis, but the changes were small and were only in some lambs compared to our previous experiments (20, 22), probably due to the eradication of parasites in most of the grazing lambs on D144 post-infection (Figure 1). Adult parasites pass into the abomasal lumen, where they feed on blood, move, and cause general histological changes, such as superficial damage to epithelial cells, hypertrophy of the mucosa, and oedema, followed by the formation of lymphoid aggregates and the infiltration of inflammatory cells in response to tissue damage (93, 94). Most of the histopathological changes in the abomasa, e.g., dilatation of glands, lymphoid aggregates near and in the *t. muscularis*, and oedema, were more notable but similar in both the CON and CHIC groups of lambs. Extensive changes in abomasal tissue previously reported, for example in infected goats, were accompanied by higher larval densities in the abomasal wall and depended on parasite number, immunology, and probably the genetic resistance of the worms (95). Our previous studies with supplementation of dry medicinal plants (*Artemisia absinthium* and *Malva sylvestris* (22) or *Althaea officinalis*, *Petasites hybridus*, *Inula helenium*, *Plantago lanceolata*, *Rosmarinus officinalis*, *Solidago virgaurea*, *Fumaria officinalis*, *Hyssopus officinalis*, and *Foeniculum vulgare* (20)) found strong local inflammation in the abomasal tissue, with the formation of lymphoid aggregates and the infiltration of immune cells in the lambs infected with *H. contortus* compared to unsupplemented infected lambs, even though the number of adult parasites in the abomasum at the end of experiments was generally highest in unsupplemented infected lambs (21, 51). The varying degrees of inflammation or changes in abomasal tissue in the CON and CHIC lambs in the present study may have been due to the delayed and incomplete defense against parasites, where a large number of parasites can cause lesions of the abomasal mucosa, which can lead to stronger local inflammation (96, 97).

## 5 Conclusion

Our results indicated that the availability of plants containing bioactive compounds in meadow grasslands may have a high potential to reduce parasite burdens in lambs and pasture contamination with infective nematode larvae which gives a future perspective in the control of parasitism in livestock. This study also performed a large-scale analysis of bioactive compounds on both pasture and the feces of lambs burdened with endoparasites, indicating their rich composition. *Haemonchus contortus* infections generally damage the abomasal mucosa, but our results suggest that bioactive plants can also likely minimise the adverse effects of GIN infection on the health of abomasal tissue.

## Data Availability

The original contributions presented in the study are included in the article/Supplementary material, further inquiries can be directed to the corresponding authors.

## Footnotes

^1^ http://www.hmdb.ca/

^2^ http://bigg.ucsd.edu/

^3^ http://pubchem.ncbi.nlm.nih.gov/

^4^ http://www.massbank.jp/

^5^ www.genome.jp

^6^ http://metlin.scripps.edu/
