# Supplementary material for: Insights into the role of bioactive plants for lambs infected with Haemonchus contortus parasite
Source: Front Vet Sci. 2025 Mar 12;12:1566720. doi: 10.3389/fvets.2025.1566720 (PMC11938065; doi:10.3389/fvets.2025.1566720)
Supplement: Supplementary file 1 [file Data_Sheet_1.PDF]

Supplementary Material

1 Supplementary Tables and Figures

Supplementary Table 1. Phytochemicals of control pasture (May-July) - Pasture I

No	Compounds	RT	UV	m/z[M-H] -	formula	MS^2 main-ion	MS^2 fragments
1	Protocatechuic acid 4-glucoside	1.3		315.0712	$C_{13}H_{16}O_9$	152.0108	
2	Vanillic acid 4-beta-D-glucoside	1.35		329.0868	$C_{14}H_{18}O_9$	167.033	283,152,123
3	Pyrogallol-2-O-glucuronide	1.5		301.0553	$C_{12}H_{14}O_9$	125.0239	168
4	trans 3-O-Caffeoylquinic acid	1.8	215,325	353.0873	$C_{16}H_{18}O_9$	191.0548	179,161,135
5	L-Tryptophan	2	279	203.0815	$C_{11}H_{12}N_2O_2$	142.066	159,116,186
6	4-Hydroxybenzoyl glucose	2.6		299.0761	$C_{13}H_{16}O_8$	137.0237	
7	trihydroxybutanoic glucoside	2.8		297.0599	$C_4H_8O_5$	135.0291	
8	5-O-p-Coumaroylquinic acid	3.2		337.0915	$C_{16}H_{18}O_8$	163.0396	191.119
9	Melilotoside	3.5		325.0915	$C_{15}H_{18}O_8$	163.0394	119
10	trans 4-O-Caffeoylquinic acid	4.4	215,325	353.0877	$C_{16}H_{18}O_9$	191.0553	179,173
11	trans 5-O-Caffeoylquinic acid	6.2	215,325	353.0882	$C_{16}H_{18}O_9$	191.0548	179,135,161
12	Derivative of Caffeoylquinic acid	6.6	215,323	533.0924		209.0293	191
13	O-p-Coumaroylquinic acid	7.30	215,325	337.0925	$C_{16}H_{18}O_8$	191.0545	
14	Luteolin 7-O-diglucuronide	7.40	289,325	637.1025	$C_{27}H_{26}O_{18}$	461.0653	285.0377
16	Vitexin xyloside	8.2	270,348	579.1353	$C_{26}H_{28}O_{15}$	489.1026	459,369,399
17	Neocarlinoside	8,4	270,348	579.135	$C_{26}H_{28}O_{15}$	459.093	489,369,399
18	Isocarlinoside	8,8	270,348	579.135	$C_{26}H_{28}O_{15}$	459.093	489,369,399
19	Isovitexin 2"-O-arabinoside (Vitexin 2"-xyloside)	8.80		563.1405	$C_{26}H_{28}O_{14}$	443	353
20	Orientin	8.90		447.093	$C_{21}H_{20}O_{11}$	357.061	327,285
21	Isovitexin 2"-O-glucoside	9.00		593.149	$C_{27}H_{30}O_{15}$	473.1074	503,383,
22	Homoplantaginin	9.1		461.1084	$C_{22}H_{22}O_{11}$	313.0338	341
23	ND	9.15		401.1813		221.1185	195
24	ND	9.25		549.1225		459.0908	489,399,369
25	ND	9.30		771.1744	$C_{36}H_{37}O_{19}$	609.1429	429,489,309
26	Isovitexin 2"-O-arabinoside (Vitexin 2"-xyloside)	9.40		563.1405	$C_{26}H_{28}O_{14}$	443.0965	353
27	D-chicoric acid	9,6	216,244,330	473.0724	$C_{22}H_{18}O_{12}$	179.0346	149,293,135,161
28	Isolariciresinol glucuronide	9.75		535.1813	$C_{26}H_{32}O_{12}$	329.1391	373,299
29	Quercetin 3,7-dirhamnoside	9.9		593.1501	$C_{27}H_{30}O_{15}$	446.0834	301
30	Quercetin 3-neohesperidoside	10.1		609.1463	$C_{27}H_{30}O_{16}$	300.0273	

31	Acteoside	10.3	215,250,329	623.1999	$C_{29}H_{36}O_{15}$	161.0239	461,315
32	ND	10.4		461.1066	$C_{22}H_{22}O_{11}$	341.0659	285,371
33	Isoacteoside	10.5	215,250,329	623.1994	$C_{29}H_{36}O_{15}$	161.0239	461,315,133
34	Azelaic acid	10.8		187.0979	$C_9H_{16}O_4$	125.0977	169,143
35	tricin glucoside	11		491.1171	$C_{23}H_{24}O_{12}$	153.0184	329
36	Tricin rutinose	11.4		637.1748	$C_{29}H_{34}O_{16}$	329.0657	

Supplementary Table 2. Phytochemicals of control pasture - (September) Pasture II

No	Compounds	RT	UV	m/z[M-H] $^-$	formula	MS^2 main-ion	MS^2 fragments
1	Geniposidic acid	1.2	245,300	373.1148	$C_{16}H_{22}O_{10}$	193.0519	149,123,167
2	Protocatechuic acid 4-glucoside	1.3		315.0712	$C_{13}H_{16}O_9$	152.0108	
3	Vanillic acid-D-glucoside	1.35	250,290	329.0884	$C_{14}H_{18}O_9$	167.0338	152,123
4	Decaffeoylverbascoside	1.4	245,292	461.1664	$C_{20}H_{30}O_{12}$	315.1079	161,135
5	8-Epiloganic acid	1.6	250,280,325	375.1259	$C_{16}H_{24}O_{10}$	169.0865	213,201,329,151
6	trans 3-O-Caffeoylquinic acid	1.8	215,325	353.0873	$C_{16}H_{18}O_9$	191.0548	179,161,135
7	L-Tryptophan	2	279	203.0815	$C_{11}H_{12}N_2O_2$	142.066	159,116,186
8	5-O-p-Coumaroylquinic acid	3.2		337.0915	$C_{16}H_{18}O_8$	163.0396	191.119
9	Pleoside	3.9		343.1032	$C_{15}H_{20}O_9$	181.0504	163,139
10	5-O-Caffeoylquinic acid	4.4	215,325	353.0878	$C_{16}H_{18}O_9$	191.0559	173
11	trans 4-O-Caffeoylquinic acid	6.2	215,325	353.0877	$C_{16}H_{18}O_9$	191.0553	179,173
12	Quercetin 3-O-glucosyl-rhamnosyl-glucoside	6.7		771.1999	$C_{33}H_{40}O_{21}$	609.1451	301,300,299
13	O-p-Coumaroylquinic acid	7.30	215,325	337.0925	$C_{16}H_{18}O_8$	191.0545	
14	Melilotoside	7.6		325.0933	$C_{15}H_{18}O_8$	163.0401	119
15	Isorhamnetin 3-sophoroside 7-rhamnoside)	8		785.215	$C_{34}H_{42}O_{21}$	623.1598	476,315,191
16	Vitexin xyloside	8.2	270,348	579.1353	$C_{26}H_{28}O_{15}$	489.1026	459,369,399
17	Luteolin C-glucoside C-xyloside	8,4	270,348	579.1375	$C_{26}H_{28}O_{15}$	459.093	489,369,399
18	Plantamajoside	8.65	216290330	639.1931	$C_{29}H_{36}O_{16}$	161.0237	179,621,459,325
19	Schaftoside	8.8	214,272,335	563.1406	$C_{26}H_{28}O_{14}$	443.0975	443,503,545
20	Isoschaftoside	8.85	214,272,335	563.1406	$C_{26}H_{28}O_{14}$	473.108	443,503,545
21	Orientin	8.9		447.0931	$C_{21}H_{20}O_{11}$	357.0614	327,429,285
22	homoplantaginin	9.1		461.1086	$C_{22}H_{22}O_{11}$	313.0338	341
23	ND	9.15		401.1813		221.1185	195
24	Quercetin 3-O-glucosyl-5-C-glucoside-rhamnosyl	9.3		771.1797	$C_{33}H_{40}O_{21}$	429	
25	Apigenin 6-C-glucoside(-6"-O-arabinoside) (Vitexin 2"-xyloside)	9.5	212,271,335	563.1424	$C_{26}H_{28}O_{14}$	413.0886	293,443

26	D-chicoric acid	9,6	216,244,330	473.0724	$C_{22}H_{18}O_{12}$	179.0346	149,293,135,161
27	Vicenin II	9.80		593.149	$C_{27}H_{30}O_{15}$	473.1074	293,503,383,
28	Lavandulifolioside	9.9	216,289,330	755.2422	$C_{34}H_{44}O_{19}$	161.0239	593,461,315
29	Isovitexin 6"-O-glucoside	9.80		593.149	$C_{27}H_{30}O_{15}$	473.1074	293,503,383,
30	Luteolin 7-O-diglucuronide	10		637.1037		285	461,161
31	Quercetin 3-neohesperidoside	10.1		609.1463	$C_{27}H_{30}O_{16}$	300.0273	
32	Hesperetin 7-(2,6-dirhamnosylglucoside)	10.2		755.2397	$C_{34}H_{44}O_{18}$	161.0244	593,461,315
33	Acteoside	10.3	215,250,329	623.1999	$C_{29}H_{36}O_{15}$	161.0239	461,315
34	Hesperetin 7-(2,6-dirhamnosylglucoside)	10.4		755.2397	$C_{34}H_{44}O_{18}$	161.0244	593,461,315
35	Malvidin	10.6		653.1738	$C_{29}H_{35}O_{17}$	329	179
36	Acteoside deriv	10.7	215,250,329	623.1999	$C_{29}H_{36}O_{15}$	161.0239	461,315
37	Tricin 7-neohesperidoside	11.3		637.1792	$C_{29}H_{34}O_{16}$	551.1741	371,174
38	Salviaflaside	12.7		521.1293	$C_{24}H_{26}O_{13}$	343.0449	359,506,
39	Myrtynoside	13		651.2283	$C_{31}H_{40}O_{15}$		

ND, not determined.

Supplementary Table 3. Phytochemicals of chicory pasture

No	Compounds	RT	UV	m/z[M-H] -	formula	MS² main-ion	MS² fragments
1	Guanosine	1		282.084	$C_{10}H_{13}N_5O_5$	150.0418	
2	Cichorioside	2.3	225,291,335	339.0728	$C_{15}H_{16}O_9$	177.019	
3	3-O-Caffeoylquinic acid	3.9	215,245,325	353.0882	$C_{16}H_{18}O_9$	191.0565	
4	5-O-Caffeoylquinic acid	4.4	215,245,325	353.0878	$C_{16}H_{18}O_9$	191.0559	
5	Cichoriin aglycon	4.5	225,290,339	177.0191	$C_9H_6O_4$	133.0285	
6	Coumarin derivative	6.4		347.0778	$C_{15}H_{14}O_4$	213.092	275,185
7	5-p-Coumaroylquinic acid	7.2	218,312	337.0927	$C_{16}H_{18}O_8$	191.0558	163
8	ND	7.3		321.098		213.092	257,185
9	4-p-Coumaroylquinic acid	8.1	219,312	337.0928	$C_{16}H_{18}O_8$	191.0559	164
10	1,3-Dicaffeoylquinic acid	8.4	216,244sh,327	515.1192	$C_{25}H_{24}O_{12}$	353.0873	191,179,135
11	1,4-Dicaffeoylquinic acid	9.2	216,244sh,327	515.1192	$C_{25}H_{24}O_{12}$	353.0873	191,179,135
12	D-chicoric acid	9,6	216,244,330	473.0724	$C_{22}H_{18}O_{12}$	179.0346	149,293,135,161
13	Quercetin 7-O-alpha-D-galactoside	10.3	256,350	463.0871	$C_{21}H_{20}O_{12}$	300.0236	301,285,241,197
14	Coumarine derivative	10.5		259.0978	$C_{15}H_{16}O_4$	215.1075	185,169,133
15	Kaempferol glucuronide	10.6	263,362	461.0643	$C_{21}H_{18}O_{12}$	285.04	
16	Apigenin glucuronide	11.5		445.0752	$C_{21}H_{18}O_{11}$	269.0433	243,183
17	Kaempferol 3-O-acetyl-glucoside	13.4	263,362	489.103	$C_{23}H_{22}O_{12}$	285.0402	284
18	ND	13.9	216265	481.114		213.0924	257,242,185,151
19	Intybin	15.3	216265	409.1296	$C_{23}H_{22}O_7$	257.0821	213,185,151

ND, not determined.

Supplementary Table 4. Phytochemicals in feces of control (CON) lambs

No	Compounds	RT	UV	m/z[M-H] -	formula	MS² main-ion	MS² fragments
1	Cumarinic acid	8		163.0369	$C_9H_8O_3$	119.0498	
2	tridec-3-enedioic acid	9		241.1438	$C_{13}H_{22}O_4$	197.1526	223
3	D-chicoric acid	9,6	216,244,330	473.0724	$C_{22}H_{18}O_{12}$	179.0346	149,293,135,161
4	Dihydrophaseic acid (DPA)	10.4		281.1389	$C_{15}H_{22}O_5$	237.1486	
5	3',4',5'-Trimethoxyflavonol	10.6		327.0877	$C_{18}H_{16}O_6$	283.0965	265,309,239
6	Azelaic acid	10.8		187.0979	$C_9H_{16}O_4$	125.0977	169,143
7	Salvigenin	10.9		327.0877	$C_{18}H_{16}O_7$	283.0965	265,309,239
8	2',3-Dihydroxy-4,4',6'-Trimethoxychalcone	11		329.1024	$C_{18}H_{18}O_6$	285.112	267,241,
9	7-Hydroxy-2',3',4'-trimethoxyisoflavan	11.2		315.122	$C_{18}H_{20}O_5$	241.1223	227,285,267,297
10	2',3-Dihydroxy-4,4',6'-Trimethoxychalcone or Melilotocarpan C	11.5		329.1031	$C_{18}H_{18}O_6$	225.0915	285,239,267,213
11	2',3-Dihydroxy-4,4',6'-Trimethoxychalcone or Diapocynin	12		329.1015	$C_{18}H_{18}O_6$	241.1234	285,267,311
12	4-Hydroxyequol	13		257.0805	$C_{15}H_{14}O_4$	213.0905	
13	Uralenneoside	13.5		285.0629	$C_{12}H_{14}O_8$	257.0676	241,221
14	9-Hydroxy-hexadecan-1,16-dioate	17.1		301.2012	$C_{16}H_{30}O_5$	283.19	265,239,221
15	Dodecanedioic acid	17.3		229.143	$C_{12}H_{22}O_4$	211.133	167
16	Epi-Lipoxin	18.1		351.2167	$C_{20}H_{32}O_5$	307.2268	333,315
17	2,3-dihydroxy-5-undecylcyclohexa-2,5-diene-1,4-dione	19.7		293.1749	$C_{17}H_{26}O_4$	221.1527	236,192
18	Tetradecanedioic acid	20.1		257.175	$C_{14}H_{26}O_4$	239.1641	195
19	3,7-Dihydroxycholan-24-oic acid	21.4		391.2851	$C_{24}H_{40}O_4$	347,	
20	7alpha-Hydroxy-3-oxo-5beta-cholan-24-oic acid	21.6		389.2686	$C_{24}H_{38}O_4$	371.2591	
21	Isodeoxycholic acid	23.1		391.2851	$C_{24}H_{40}O_4$	347,	

Supplementary Table 5. Phytochemicals in feces of experimental (CHIC) lambs

No	Compounds	RT	UV	m/z[M-H] -	formula	MS² main-ion	MS² fragments
1	ND	6.5		297.1348	$C_{15}H_{22}O_6$	253.1437	191
2	Gallic acid octyl ester	7		281.1389	$C_{15}H_{22}O_5$	237.1486	
3	Gallic acid octyl ester	7.5		281.1391	$C_{15}H_{22}O_5$	237.1486	
4	ND	7.9		297.1348	$C_{15}H_{22}O_6$	253.1437	191
5	Cumarinic acid	8		163.0369	$C_9H_8O_3$	119.0498	
6	(S)-2,3-Dihydro-7-hydroxy-2-methyl-4-oxo-4H-1-benzopyran-5-acetic acid.	8.6		235.0608	$C_{12}H_{12}O_5$	191.0709	149

7	ND	8.8		243.1602		183.1381	
8	3-Methoxyphenylacetic acid	8.9		165.0569	$C_9H_{10}O_3$	147.0449	
9	tridec-3-enedioic acid	9		241.1438	$C_{13}H_{22}O_4$	197.1526	223
10	pentadeca-4,7,10-trienedioic acid	9.5		265.1437	$C_{15}H_{22}O_4$	161.0968	191,247,221,203
11	D-chicoric acid	9,6	216,244,330	473.0724	$C_{22}H_{18}O_{12}$	179.0346	149,293,135,161
12	ND	10.4		265.1437	$C_{15}H_{22}O_4$	161.0968	191,247,221,203
13	2',7-Dihydroxy-4',6-dimethoxyisoflavan	10.5		301.1077	$C_{17}H_{18}O_5$	257.1178	227,198,211
14	3',4',5'-Trimethoxyflavonol	10.6		327.0877	$C_{18}H_{16}O_6$	283.0965	265,309,239
15	Salvigenin	10.8		327.0877	$C_{18}H_{16}O_7$	283.0965	265,309,239
16	2',3-Dihydroxy-4,4',6'-Trimethoxychalcone	10.9		329.1024	$C_{18}H_{18}O_6$	285.112	267,241,
17	7-Hydroxy-2',3',4'-trimethoxyisoflavan	11.2		315.122	$C_{18}H_{20}O_5$	241.1223	227,285,267,297
18	2',3-Dihydroxy-4,4',6'-Trimethoxychalcone or Melilotocarpan C	11.5		329.1031	$C_{18}H_{18}O_6$	225.0915	285,239,267,213
19	2',3-Dihydroxy-4,4',6'-Trimethoxychalcone or Diapocynin	12		329.1015	$C_{18}H_{18}O_6$	241.1234	285,267,311
20	Dihydrophaseic acid	12.5		281.1388	$C_{15}H_{22}O_5$	237.149	219
21	4-Hydroxyequol	13		257.0812	$C_{15}H_{14}O_4$	213.0905	
22	Uralenneoside	13.5		285.0635	$C_{12}H_{14}O_8$	257.0679	241,221,183
23	Dodecanedioic acid	17.3		229.1448	$C_{12}H_{22}O_4$	211.133	167
24	2,3-dihydroxy-5-undecylcyclohexa-2,5-diene-1,4-dione	19.7		293.1749	$C_{17}H_{26}O_4$	221.1527	236,192
25	Tetradecanedioic acid	20.1		257.175	$C_{14}H_{26}O_4$	239.1641	
26	3,7-Dihydroxycholan-24-oic acid	21.4		391.2851	$C_{24}H_{40}O_4$	347,	
27	7alpha-Hydroxy-3-oxo-5beta-cholan-24-oic acid	21.6		389.2686	$C_{24}H_{38}O_4$	371.2591	
28	Isodeoxycholic acid	23.1		391.2851	$C_{24}H_{40}O_4$	347,	

ND, not determined.

1.1 Supplementary Figures

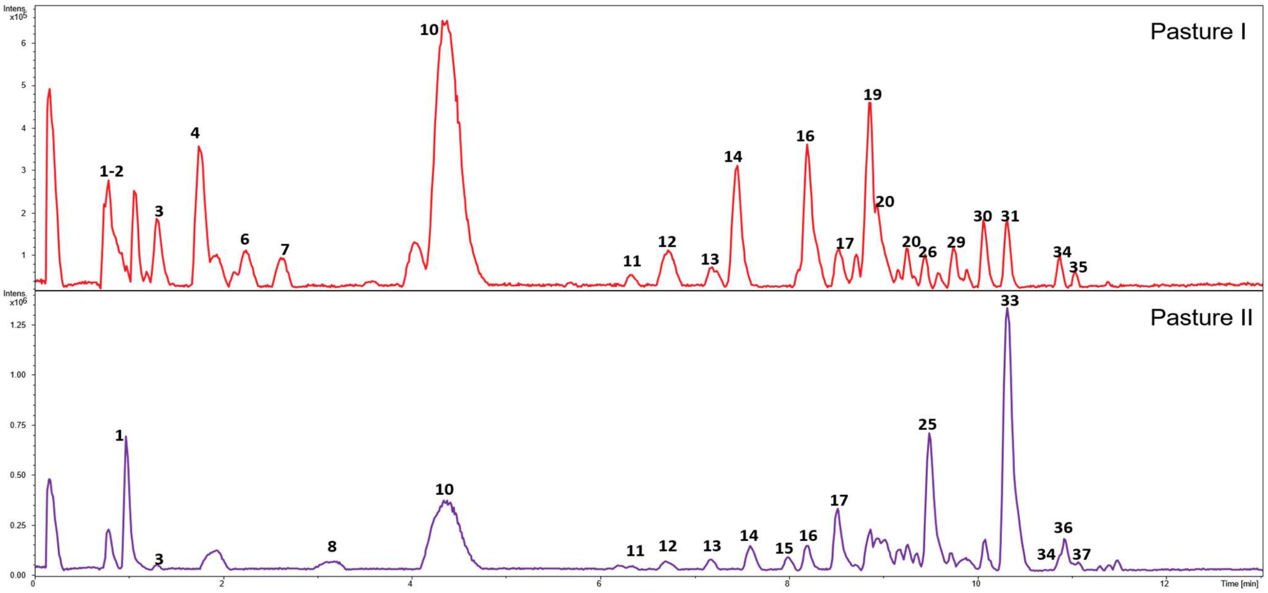

Supplementary Figure 1. Base Peak Chromatogram of Pasture I and Pasture II with main compounds. Peak numbers in Fig. 1 represent the main compounds as numbered in Tables 1 and 2.

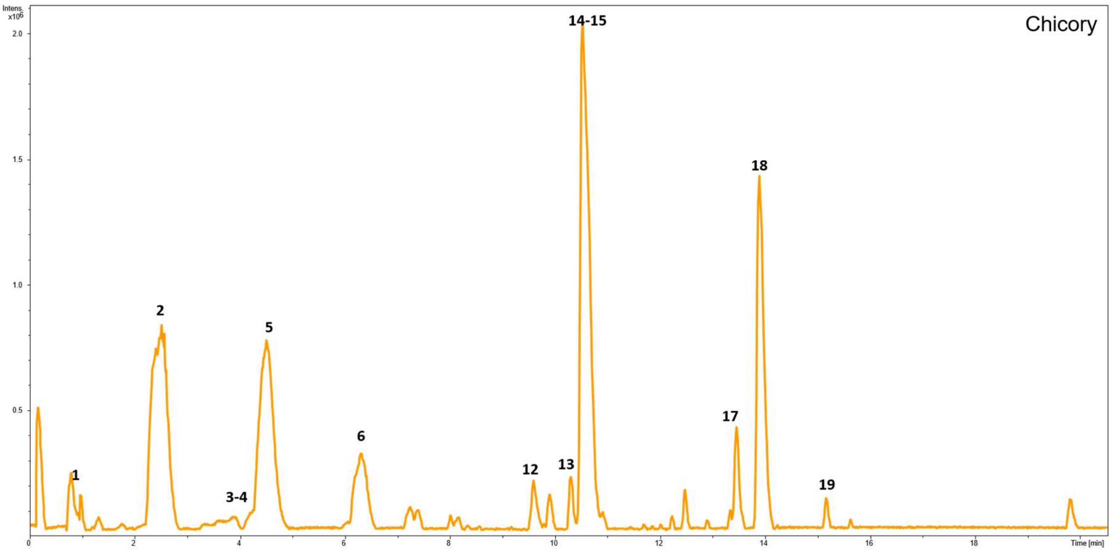

Supplementary Figure 2. Base Peak Chromatogram of Chicory with main compounds. Peak numbers in Fig. 2 represent the main compounds as numbered in Table 3.
